# Lipid metabolic reprogramming in osteoarthritis: cartilage-centered mechanisms, whole-joint interactions, and therapeutic challenges

**DOI:** 10.3389/fendo.2026.1825725

**Published:** 2026-06-29

**Authors:** Kai Xu, Junchen Li, Genghong Wang, Jing Lin, Ji Li

**Affiliations:** 1 Heilongjiang University of Chinese Medicine, Harbin, China; 2 Second Affiliated Hospital of Heilongjiang University of Chinese Medicine, Harbin, China; 3 The Second Affiliated Hospital of Harbin Medical University, Harbin, China

**Keywords:** ferroptosis, infrapatellar fat pad, lipid metabolism, lipotoxicity, osteoarthritis

## Abstract

Osteoarthritis (OA) is a biologically heterogeneous whole-joint disorder characterized by cartilage damage, synovitis, subchondral bone remodeling, pain, and progressive functional decline. Although OA has long been viewed primarily as a mechanically driven disorder, clinical and experimental evidence indicates that lipid metabolic dysregulation contributes to OA susceptibility, symptom burden, and disease progression in at least a subset of patients. This review focuses on how lipid metabolic dysregulation shapes inflammatory, catabolic, and survival pathways in OA, with particular emphasis on cartilage-centered mechanisms and whole-joint metabolic interactions. We discuss cholesterol dysregulation, fatty-acid lipotoxicity, neutral lipid/lipid-droplet accumulation, phospholipid remodeling, and obesity-associated adipose-joint crosstalk as major components of metabolic OA. Key pathways linking lipid imbalance to OA pathology include the CH25H-CYP7B1-RORα axis, fatty-acid-induced lipotoxic stress, mitochondrial oxidative injury, and phospholipid peroxidation-driven ferroptosis. Under conditions of iron dyshomeostasis, lipid peroxidation can compromise chondrocyte membrane integrity and promote ferroptotic cell death, thereby amplifying matrix-destructive responses. HIF-1α signaling is context-dependent: basal or appropriately stabilized HIF-1α may support hypoxic adaptation and mitochondrial quality control, whereas lipid overload, lactate accumulation, and chronic inflammation can redirect HIF-1α toward proinflammatory metabolic amplification. We also evaluate the translational prospects and current limitations of lipid-targeted strategies, including statins, metabolic interventions, and nutritional approaches. Current evidence supports metabolic stratification in OA, but important gaps remain in linking circulating lipid alterations to tissue-specific mechanisms and in identifying patient subsets most likely to benefit from lipid-targeted intervention.

## 1 Introduction

Osteoarthritis (OA) is the most prevalent chronic joint disease and is now widely recognized as a biologically heterogeneous whole-joint disorder rather than a disease confined to articular cartilage alone (1–3). According to the Global Burden of Disease Study 2021, approximately 595 million people were living with OA in 2020, and the global number of affected individuals is projected to approach 1 billion by 2050, indicating that OA will remain a major source of disability and health-care expenditure in aging populations (4).

This disease is characterized not only by cartilage erosion, but also by synovitis, subchondral bone remodeling, osteophyte formation, pain sensitization, and progressive loss of joint function, which together support the concept of OA as a failure of joint homeostasis at multiple tissue levels (1). Although mechanical overload remains a major risk factor, it is no longer sufficient to explain the full spectrum of OA phenotypes, because aging, obesity, low-grade inflammation, and metabolic dysfunction also shape disease initiation and progression (1, 5–8).

Among these non-mechanical drivers, lipid metabolism represents a testable pathogenic axis in OA (9–13). Disturbances in cholesterol handling, fatty-acid exposure, and phospholipid composition have been linked to chondrocyte injury, extracellular matrix turnover, and inflammatory signaling in OA-relevant tissues (14–18). Lipidomic and metabolomic studies further suggest that specific lipid signatures are associated with OA severity or advanced knee OA (19, 20). These findings are consistent with broader evidence linking dyslipidemia and bioactive lipids to OA risk and pathobiology (13, 21). This review therefore examines OA-specific lipid metabolic dysregulation with a focus on cartilage-centered mechanisms, whole-joint metabolic interactions, and current translational limits.

### 1.1 Literature search and evidence selection

This narrative review was based on a structured literature search of PubMed, Web of Science, and Scopus. The main search terms included “osteoarthritis”, “lipid metabolism”, “cholesterol”, “fatty acid”, “lipotoxicity”, “lipid droplet”, “phospholipid”, “ferroptosis”, “HIF-1α”, “infrapatellar fat pad”, and “metabolic osteoarthritis”. Original experimental studies, human lipidomic or metabolomic studies, imaging-based cohort studies, and mechanistic studies using chondrocytes, synovial tissues, infrapatellar fat pad, or animal models were prioritized. Review articles were used mainly for background framing and were not used as the primary source for specific mechanistic claims when original evidence was available. All references were manually checked for bibliographic accuracy, relevance to the cited statement, and consistency between the in-text citation and the reference list.

## 2 Osteoarthritis as a metabolically responsive whole-joint disease

The view that OA is metabolically responsive is supported by epidemiological, MRI-based, and molecular profiling evidence (5–8, 10, 22). In particular, MRI-based evidence has shown that metabolic syndrome is related to structural progression in knee OA even after adjustment for conventional clinical variables (22). This observation is consistent with broader epidemiological and conceptual work linking metabolic syndrome to OA risk, symptom burden, and phenotype definition (7, 23–27).

These findings indicate that OA in at least a subset of patients is shaped by systemic metabolic abnormalities acting through endocrine, inflammatory, and lipid-mediated mechanisms, rather than by load alone (5, 6, 8, 9, 22). Lipidomic profiling has added another layer of evidence by showing that OA severity is associated with specific changes in higher-order lipids and oxylipins, although the proportion of variance explained by circulating lipids remains modest (20). These data fit with broader discussions of bioactive lipids and dyslipidemia in OA (13, 21).

Similarly, the plasma lysophosphatidylcholine-to-phosphatidylcholine ratio has been reported to predict advanced knee OA, indicating that lipid composition, not only total lipid burden, may carry clinically relevant information (19). These findings support a whole-joint view of metabolic OA in which disturbed lipid handling extends beyond chondrocytes to involve synovium, subchondral bone, and periarticular adipose tissues (1–3, 6, 10).

Among these compartments, adipose-associated signals are particularly relevant because obesity-related lipid excess can shape the inflammatory and metabolic state of the joint through adipokines, free fatty acids, and local tissue crosstalk (28–34). This mechanism is particularly evident in the infrapatellar fat pad and related periarticular adipose tissues, which provide a biologically plausible link between systemic metabolic stress and local cartilage injury (30, 35–38).

These observations should nevertheless be interpreted cautiously, because serum or plasma lipid signatures do not directly replicate the biochemical environment of the joint cavity, and human association studies cannot by themselves establish causal pathways at the tissue level (20, 22). The adipose-joint axis should therefore be interpreted alongside mechanistic evidence from experimental models and cell-based studies. These considerations position obesity-associated adipose-joint crosstalk as a useful framework for linking systemic metabolic dysfunction to cartilage-centered lipid metabolic reprogramming.

### 2.1 Obesity-associated adipose-joint crosstalk

Obesity contributes to osteoarthritis not only by increasing mechanical load, but also by establishing a metabolic and inflammatory milieu that alters the biology of the whole joint (5, 6, 25, 26). Metabolic syndrome has been associated with structural progression on knee MRI, supporting the existence of a metabolically responsive OA phenotype rather than a purely load-driven disorder (7, 22, 23). In this context, hypertrophic adipose tissue functions as an active endocrine and immunometabolic organ rather than a passive energy reservoir (31–33). Adipocyte hypertrophy is usually accompanied by adipose-tissue hypoxia, macrophage infiltration, enhanced lipolysis, and adipokine imbalance. These changes increase the systemic availability of free fatty acids and inflammatory mediators, which may expose chondrocytes to a lipid-rich and cytokine-rich milieu. Under this condition, chondrocytes may shift from adaptive lipid handling toward lipotoxic stress, lipid-droplet accumulation, mitochondrial dysfunction, and inflammatory catabolic signaling. Excess nutrient supply and adipocyte dysfunction promote the release of free fatty acids, adipokines, and inflammatory mediators into the circulation, thereby increasing the likelihood that systemic metabolic stress is translated into local joint injury (31–34). In addition, dietary fat-associated OA chondrocytes show altered lipid-droplet handling and adaptive responses to lipotoxic stress, suggesting that chronic exposure to excess lipids can reprogram cartilage metabolism itself (39).

Within the knee joint, the infrapatellar fat pad (IFP) is a major local adipose structure linking systemic metabolic imbalance to tissue-specific OA pathology (30, 35–38, 40). The IFP should not be regarded as a merely structural or inert adipose compartment (30, 35–37). It lies in close anatomical continuity with the synovium and is positioned to influence cartilage, synovial fibroblasts, and resident immune cells through paracrine signaling (30, 35–37). Klein-Wieringa et al. demonstrated that the IFP of patients with OA has an inflammatory phenotype and releases higher levels of mediators such as IL-6, adipsin, and adiponectin than paired subcutaneous adipose tissue, indicating that the local adipose compartment is biologically distinct and potentially pathogenic in OA (28). This interpretation is consistent with the view that the IFP acts as an active intra-articular adipose tissue rather than an inert bystander (29). Single-cell transcriptomic analysis provides tissue-resolved evidence for this adipose-synovial unit (41). Tang et al. showed that the IFP and synovium form an integrated tissue unit with shared mesenchymal lineage relationships and OA-associated inflammatory and profibrotic remodeling. Their study also identified APOE signaling as a potentially relevant pathway in this tissue niche, and soluble APOE accelerated cartilage proteoglycan loss ex vivo, further linking adipose-synovial alterations to cartilage damage (41).

The adipose-joint axis is better interpreted as a disease primer than as an isolated trigger (42). In a mouse study, high-fat feeding alone did not induce frank inflammation or fibrosis in the infrapatellar fat pad, but when combined with surgically induced joint injury it promoted pathologic IFP changes and a more progressive OA state (42). These data argue against a simplistic model in which obesity alone is sufficient to explain all metabolic OA changes. Current evidence suggests that obesity-associated OA is shaped by coordinated crosstalk among systemic adipose tissue, the infrapatellar fat pad, synovium, and cartilage (28, 29, 33, 38, 41). Adipose-joint interactions should therefore be incorporated into metabolic OA phenotyping and into experimental designs that test tissue-specific mechanisms (5, 6, 25, 26).

**Figure 1:**
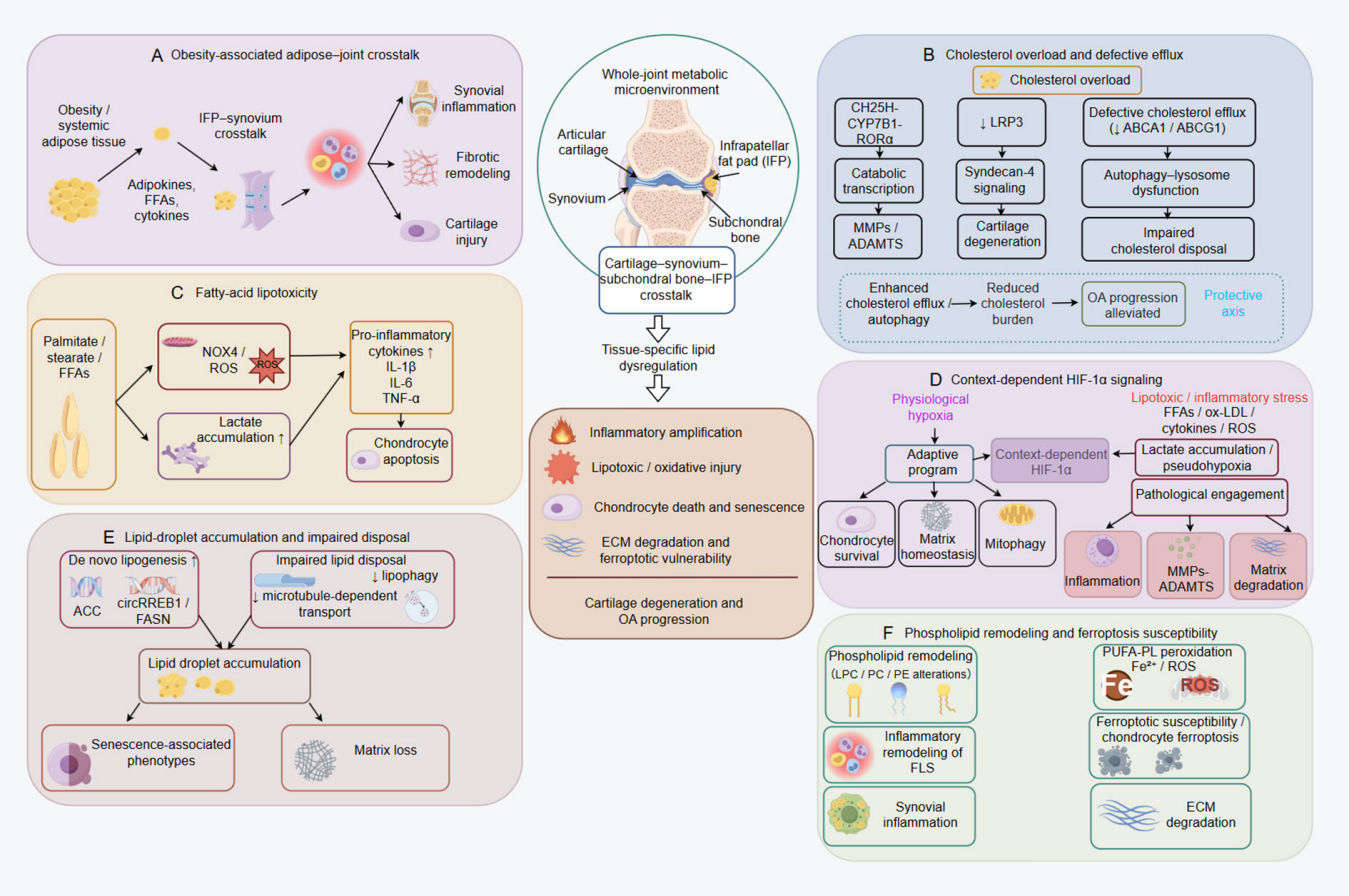
Lipid metabolic reprogramming in osteoarthritis: systemic metabolic stress, whole-joint crosstalk, and chondrocyte injury. **(A)** Obesity-associated adipose–joint crosstalk. **(B)** Cholesterol overload and defective efflux. **(C)** Fatty-acid lipotoxicity. **(D)** Context-dependent HIF-1α signaling. **(E)** Lipid-droplet accumulation and impaired disposal. **(F)** Phospholipid remodeling and ferroptosis susceptibility. This schematic illustrates how systemic metabolic stress, including obesity, metabolic syndrome, and dyslipidemia, may be translated into osteoarthritis-related joint pathology through circulating lipid signals, adipokines, free fatty acids, and local adipose–joint crosstalk. Within the joint microenvironment, the infrapatellar fat pad, synovium/fibroblast-like synoviocytes, cartilage, and subchondral bone form an interactive metabolic niche. In chondrocytes, lipid metabolic dysregulation involves cholesterol overload and defective efflux, fatty-acid lipotoxicity, neutral lipid/lipid-droplet stress, phospholipid remodeling, ferroptosis susceptibility, and context-dependent HIF-1α signaling. These processes converge on inflammatory amplification, redox injury, apoptosis, senescence, ferroptosis, extracellular matrix degradation, cartilage degeneration, and whole-joint progression. Solid arrows indicate mechanistic directionality. FFAs, free fatty acids; FLS, fibroblast-like synoviocytes; IFP, infrapatellar fat pad; LPC, lysophosphatidylcholine; MetS, metabolic syndrome; PC, phosphatidylcholine; TG, triglyceride.

The major lipid-associated pathways discussed in this review and their relationships to cartilage injury, inflammatory signaling, senescence, and matrix degradation are summarized in **Figure 1**.

## 3 Lipid metabolic reprogramming in osteoarthritic cartilage

As outlined in **Figure 1**, lipid dysregulation in OA is a coordinated process rather than a single-pathway event. Against this whole-joint background, cartilage remains the tissue in which these abnormalities can be linked most directly to defined catabolic and survival pathways. The following sections therefore focus on cholesterol handling, fatty-acid lipotoxicity, mitochondrial dysfunction and oxidative stress, neutral-lipid storage, phospholipid remodeling, and hypoxia-related metabolic adaptation in osteoarthritic chondrocytes.

### 3.1 Cholesterol influx/efflux imbalance and chondrocyte catabolism

Among the lipid pathways implicated in OA, cholesterol metabolism is supported by several OA-specific mechanistic studies, particularly those addressing the CH25H-CYP7B1-RORα axis and the cholesterol-LRP3-Syndecan-4 pathway (14, 15). Reviews place cholesterol metabolism within the broader lipid-related pathobiology of OA (43, 44). Identification of the CH25H-CYP7B1-RORα axis established a direct mechanistic link between cholesterol metabolism and catabolic transcriptional programs in OA chondrocytes (14).

Subsequent work further showed that cholesterol-induced downregulation of low-density lipoprotein receptor-related protein 3 (LRP3) promotes cartilage degeneration by targeting Syndecan-4, thereby linking cholesterol overload to a specific degenerative axis in articular cartilage (15). Experimental data also indicate that high LDL conditions enhance synovial activation and accelerate ectopic bone formation in osteoarthritis models, suggesting that excessive cholesterol burden is relevant not only to chondrocytes but to the broader joint microenvironment (45). At the cellular level, oxidized LDL has been shown to disrupt TFEB-regulated autophagy-lysosome activity in chondrocytes, thereby accelerating inflammatory cell death and matrix-destructive responses under OA-like conditions (46).

Conversely, enhancing intracellular cholesterol efflux in chondrocytes alleviates OA progression, indicating that the critical pathogenic event is not simply the presence of cholesterol, but the failure of local cholesterol processing and disposal within cartilage (47).

High cholesterol has been reported to suppress circARPC1B-related protective regulation and destabilize cytoskeletal and matrix-supporting pathways in articular cartilage (48). Resveratrol-mediated activation of the SIRT1/FoxO1 pathway has been reported to improve cholesterol metabolism and attenuate OA-like changes in experimental settings (49).

Available experimental evidence suggests that OA-associated cholesterol injury involves excessive influx, impaired efflux, disturbed intracellular trafficking, and secondary failure of autophagic quality control, which together contribute to extracellular matrix breakdown and cartilage degeneration (14, 15, 45–47).

### 3.2 Fatty-acid overload, lipotoxicity, and redox injury

Fatty-acid dysregulation in OA cannot be reduced to a simple quantitative model in which higher lipid burden uniformly translates into greater inflammation (50, 51). The strongest detrimental evidence concerns saturated fatty acids, which can induce lipotoxic stress in chondrocytes and amplify existing inflammatory responses (16, 52). Alvarez-Garcia et al. demonstrated that palmitate has proapoptotic and proinflammatory effects on articular cartilage and acts synergistically with IL-1β to drive chondrocyte death and cartilage matrix breakdown, indicating that saturated fatty acids can intensify classic OA cytokine injury rather than merely coexist with it (16). Miao et al. further showed that stearic acid increases the production of IL-1β, IL-6, and TNF-α partly through the lactate-HIF-1α pathway, thereby connecting fatty-acid overload with metabolic rewiring and inflammatory amplification in chondrocytes (52).

In parallel, oleic/palmitate exposure has been reported to enhance NOX4 expression, increase ROS production, and promote apoptosis in human articular chondrocytes, highlighting the role of redox injury in fatty-acid-mediated cartilage damage (53). These studies indicate that fatty acids influence inflammatory cytokine output, redox balance, and cell-survival pathways within cartilage (16, 52, 53).

By contrast, omega-3 polyunsaturated fatty acids have been discussed as anti-inflammatory and potentially protective lipid mediators in OA (54, 55). A recent meta-analysis indicated that n-3 PUFA supplementation can relieve pain and improve joint function in patients with OA, although this does not yet amount to proof of robust structure-modifying efficacy (56).

Current data indicate that fatty-acid imbalance in OA is best understood as lipotoxic signaling and redox injury, with clinical benefit from fatty-acid modification remaining modest and context-dependent (50, 51, 54, 56).

### 3.3 Mitochondrial dysfunction and oxidative stress as convergence nodes of lipid-mediated cartilage injury

Mitochondrial dysfunction represents a major convergence point through which lipid overload is translated into chondrocyte injury. Saturated fatty-acid exposure, oxidized lipoproteins, and lipid-derived oxidative stress can disturb mitochondrial quality control, increase mitochondrial ROS production, and impair the balance between adaptive energy metabolism and catabolic activation (46, 53, 70). In osteoarthritic chondrocytes, mitochondrial ROS may amplify inflammatory cytokine production, matrix-degrading enzyme expression, apoptotic susceptibility, and lipid peroxidation-dependent ferroptotic vulnerability (52, 53, 60, 63). This process is mechanistically linked to defective mitophagy and impaired intracellular lipid disposal, suggesting that mitochondrial dysfunction is not merely a downstream consequence of OA but an active amplifier of lipid-mediated cartilage degeneration (59, 70).

### 3.4 Neutral-lipid/lipid-droplet accumulation and chondrocyte senescence

Neutral lipid-related pathology in OA is better understood as abnormal neutral-lipid storage within cartilage than as a simple reflection of circulating neutral lipid concentration (57, 58). In an obesity-associated mouse model, Liu et al. showed that cartilage itself accumulates lipids and that this process is associated with increased acetyl-CoA carboxylase-mediated *de novo* lipogenesis, suggesting that metabolically stressed cartilage becomes an active site of lipid synthesis and deposition (57). These findings argue against the simplistic assumption that lipids appearing in cartilage are passively delivered from the circulation without local metabolic remodeling. At the cellular level, Gong et al. showed that CircRREB1 mediates lipid metabolism-related senescent phenotypes in chondrocytes through post-translational regulation of FASN, thereby directly linking fatty-acid synthesis machinery to chondrocyte senescence and OA progression (58). Lipid synthesis, rather than lipid uptake alone, may contribute to senescence-associated cartilage degeneration and may help explain why lipid-rich chondrocytes display senescence-related pathological features (58).

More recently, Yu et al. demonstrated that aberrant stress impairs microtubule-dependent intracellular transport, blocks lipophagy, and causes lipid-droplet accumulation in chondrocytes, providing a mechanistic basis for the persistence of stored lipids in diseased cartilage (59).

Persistent lipid-droplet accumulation may sustain lipid-storage stress, impair intracellular transport, and reinforce senescence-associated phenotypes in chondrocytes (58, 59). The neutral lipid axis in OA is therefore best discussed in terms of lipid-droplet dynamics, *de novo* lipogenesis, and impaired lipophagy, rather than as a generic reflection of serum neutral lipids (57–59).

### 3.5 Phospholipid remodeling and ferroptosis susceptibility

Phospholipid metabolism is relevant to OA because phospholipids are structural membrane components, precursors of bioactive inflammatory mediators, and potential substrates for oxidative lipid injury (13, 17, 18). A lipidomic study of synovial fluid demonstrated that the composition of phospholipid species is altered in human OA knee joints, supporting the view that phospholipid remodeling is part of the diseased joint environment rather than an incidental laboratory finding (17).

Separately, plasma lysophosphatidylcholine-to-phosphatidylcholine ratios have been reported to predict advanced knee OA, suggesting that phospholipid-derived systemic signatures may carry clinically relevant information (19). Proteomic and lipidomic analyses of fibroblast-like synoviocytes indicate that LPC-rich stimulation modifies synovial-cell signaling in OA-relevant contexts (18).

OA-focused reviews frame ferroptosis mainly as an iron-dependent lipid peroxidation process linked to chondrocyte injury, but disease-specific causal claims should still rely primarily on experimental chondrocyte and animal-model studies (60–62). Experimental work has shown that chondrocyte ferroptosis contributes to OA progression and matrix-destructive responses; inhibition of ferroptotic injury attenuated chondrocyte damage *in vitro* and reduced cartilage degeneration in experimental models (63, 64). Mechanistically, ferroptotic chondrocyte death is driven by the convergence of iron dyshomeostasis and lipid peroxide accumulation. Excess labile iron can facilitate ROS generation and promote the peroxidation of phospholipids enriched in polyunsaturated fatty-acid chains. When antioxidant defenses such as the cystine-glutathione-GPX4 axis are insufficient, lipid peroxides accumulate within cellular membranes, causing membrane instability, loss of redox homeostasis, and ferroptotic chondrocyte death. In OA, this mechanism provides a plausible link between phospholipid remodeling, oxidative stress, and matrix-destructive cartilage degeneration, although direct lipid species-level evidence in human cartilage remains incomplete.

However, direct evidence linking specific phospholipid species to ferroptotic susceptibility in human OA cartilage remains limited, and much of the current interpretation is extrapolated from the biochemical requirement of lipid peroxidation in ferroptosis and from experimental chondrocyte models. Thus, phospholipid abnormalities in OA should currently be viewed as both candidate biomarkers and plausible contributors to synovial-cell inflammatory remodeling and chondrocyte ferroptotic vulnerability, rather than as fully validated causal drivers across all OA phenotypes (17, 18, 63).

### 3.6 HIF-1α-centered metabolic adaptation and cartilage homeostasis

HIF-1α occupies a special position in cartilage biology because articular cartilage is physiologically hypoxic and basal hypoxia signaling supports chondrocyte survival and tissue homeostasis (65–69). HIF-1α should not be described simply as a harmful factor in OA, because part of its function is adaptive under physiological cartilage hypoxia (65, 66, 68). Because HIF-1α and HIF-2α represent distinct hypoxia-responsive regulatory axes in cartilage, this section focuses on HIF-1α as a lipid-stress-sensitive node connecting hypoxic adaptation, mitochondrial quality control, and proinflammatory metabolic amplification. HIF-2α, although highly relevant to OA catabolism, is acknowledged as a separate regulatory axis and is not discussed in detail here.

However, this adaptive program can be distorted under conditions of lipid overload and chronic inflammatory stress, where HIF-1α becomes integrated into pathogenic metabolic circuits rather than preserving homeostasis (52, 70). Miao et al. showed that stearic acid-induced inflammatory cytokine production in chondrocytes is mediated partly through activation of the lactate-HIF-1α pathway, indicating that HIF-1α can be recruited into a lipotoxic proinflammatory response when substrate excess and metabolic disturbance are present (52). At the same time, stabilization of HIF-1α has been reported to alleviate OA by enhancing mitophagy, suggesting that appropriate HIF-1α signaling may help preserve mitochondrial quality control and limit tissue injury under certain conditions (70). These observations suggest that the biological output of HIF-1α depends on context, including the balance among hypoxic adaptation, mitochondrial stress, lactate accumulation, and inflammatory signaling (65, 70).

In OA, HIF-1α is better framed as a nodal regulator at the interface between metabolic adaptation and disease amplification, rather than as a one-directional marker of either protection or damage (52, 65–68, 70). To integrate the mechanisms discussed across Sections 2.1 and 3.1-3.6, the major lipid-associated abnormalities, representative pathways, and their principal pathogenic consequences in OA are summarized in **Table 1**.

**Table 1:** Major lipid metabolic abnormalities, representative pathways, and pathogenic consequences in osteoarthritis.

Pathway/biological domain	Representative molecules or axis	Major tissue/compartment	Main pathogenic consequence in OA	Key biological manifestations	Key references
Cholesterol overload and defective efflux	CH25H-CYP7B1-RORα; LRP3; ox-LDL; TFEB; circARPC1B; SIRT1/FoxO1	Articular cartilage; synovium	Catabolic transcription, cartilage degeneration, inflammatory cell death, ectopic bone formation	Increased catabolic enzyme expression, impaired cholesterol clearance, disrupted autophagy-lysosome function, inflammatory mediator release	(14, 15, 45–49)
Fatty-acid lipotoxicity	Palmitate; stearate; FFAs; NOX4/ROS; lactate-HIF-1α	Chondrocytes; cartilage microenvironment	Inflammatory amplification, redox injury, apoptosis	Increased IL-1β, IL-6, and TNF-α; ROS accumulation; impaired cell-survival pathways	(16, 52, 53)
Omega-3 fatty-acid modulation	n-3 PUFAs	Systemic intervention; clinical symptom domain	Modulation of inflammatory tone and symptoms	Potential anti-inflammatory and symptom-relieving effects; structural modification remains unproven	(54–56)
Neutral-lipid/lipid-droplet accumulation and stress	TGs; ACC-mediated *de novo* lipogenesis; circRREB1/FASN; lipid droplets	Chondrocytes; cartilage	Chondrocyte senescence, matrix loss, and impaired lipid handling	Cartilage lipid accumulation, *de novo* lipogenesis, FASN-related senescent phenotypes, impaired lipid clearance	(57–59)
Defective lipophagy and lipid disposal	Lipid droplets; microtubule-dependent transport machinery	Chondrocytes	Persistence of stored lipids and lipid-storage stress	Impaired intracellular transport, defective lipid clearance, persistent metabolic stress	(59)
Phospholipid remodeling	LPC; PC; PE	Synovial fluid; fibroblast-like synoviocytes; cartilage	Altered phospholipid composition and synovial-cell inflammatory remodeling	Altered phospholipid composition, lysophospholipid enrichment, inflammatory remodeling of synovial cells	(17–19)
Ferroptosis susceptibility linked to phospholipid imbalance	Lipid peroxidation-prone phospholipids; iron-dependent lipid peroxidation	Chondrocytes	Ferroptotic cell death and matrix degradation	Iron-dependent lipid peroxidation, increased matrix injury, susceptibility to ferroptotic injury	(60–64)
Hypoxia-related metabolic adaptation	HIF-1α; lactate-HIF-1α; mitophagy	Chondrocytes	Context-dependent switch between adaptive survival and inflammatory amplification	Homeostatic support under physiological hypoxia; inflammatory amplification under lipotoxic stress	(65–70)
Obesity-associated adipose-joint crosstalk	FFAs; adipokines; infrapatellar fat pad-derived mediators; APOE-related signaling	Systemic adipose tissue; infrapatellar fat pad; synovium	Joint priming, inflammation, fibrosis, cartilage injury	Adipokine release, inflammatory adipose phenotype, integrated IFP-synovium remodeling	(28–42)
Mitochondrial dysfunction and oxidative stress	mtROS; impaired mitophagy; NOX4/ROS; lipid peroxide stress	Chondrocytes; cartilage microenvironment	Redox amplification, apoptosis, ferroptosis susceptibility, matrix degradation	Increased ROS/mtROS, impaired mitochondrial quality control, reduced stress tolerance, enhanced inflammatory catabolism	(46, 52, 53, 60, 63, 70)

This table summarizes the principal lipid-associated abnormalities discussed in this review and shows how distinct lipid classes or pathways converge on recurrent pathological outputs in osteoarthritis, including inflammatory amplification, mitochondrial oxidative stress, redox injury, senescence, ferroptosis susceptibility, and extracellular matrix loss.

CH25H, cholesterol 25-hydroxylase; FFAs, free fatty acids; HIF-1α, hypoxia-inducible factor-1α; IFP, infrapatellar fat pad; LPC, lysophosphatidylcholine; LRP3, low-density lipoprotein receptor-related protein 3; ox-LDL, oxidized low-density lipoprotein; PC, phosphatidylcholine; PE, phosphatidylethanolamine; PUFAs, polyunsaturated fatty acids; ROS, reactive oxygen species; TFEB, transcription factor EB; TGs, triglycerides.

## 4 Discussion and future perspectives

### 4.1 Why clinical translation remains inconsistent

Lipid metabolism is a biologically plausible therapeutic axis in OA, but consistent disease-modifying effects have not yet been established (71–73). Statin studies illustrate the limited translation from biological plausibility to reproducible structural benefit (71, 72). In the Rotterdam Study, statin use was associated with reduced progression of knee OA, suggesting that cholesterol-lowering or pleiotropic anti-inflammatory effects may influence structural outcomes in some patient populations (71). Several factors may explain the inconsistent clinical outcomes of statins and other lipid-targeted therapies in OA. First, systemic LDL lowering does not necessarily restore tissue-specific cholesterol efflux, lipid trafficking, or lipid-droplet disposal within chondrocytes and synovial cells. Second, lipid-lowering treatment may be stage-dependent, with greater potential to modulate early inflammatory responses than to reverse established cartilage loss or subchondral remodeling. Third, unselected OA populations are biologically heterogeneous; therefore, any benefit in metabolically driven OA may be diluted in cohorts dominated by trauma, malalignment, or advanced mechanical failure. Finally, pain relief, synovial inflammation, and structural modification are distinct therapeutic outcomes and should not be interpreted as interchangeable endpoints.

However, experimental work in dyslipidemic mice showed that intensive cholesterol-lowering treatment reduced synovial inflammation in early collagenase-induced OA but did not reduce pathology at end-stage disease, indicating that attenuation of inflammation does not necessarily translate into durable structural modification (72). The same caution applies to fatty-acid-directed therapy, because although n-3 PUFA supplementation has shown efficacy for relieving pain and improving joint function in meta-analysis, the available evidence does not yet establish a robust structure-modifying effect in OA (56). Consistent with this uncertainty, current OA guidelines still prioritize exercise, education, and weight management as core treatment components, whereas lipid-targeted pharmacotherapy has not yet entered routine OA management (74).

The recent semaglutide trial in persons with obesity and knee OA is especially informative in this regard, because it showed that metabolic intervention can significantly reduce body weight and improve OA-related pain, thereby strengthening the clinical relevance of the metabolic phenotype without proving direct reversal of cartilage pathology (73). These data suggest that future lipid-focused OA therapy will likely require better patient stratification, because a metabolically driven OA endotype may respond differently from OA dominated by trauma, malalignment, or advanced mechanical failure (7, 20, 22, 75). The next phase of translational work should integrate lipidomics, imaging, and clinical phenotyping so that symptomatic improvement, inflammatory modulation, and structural modification are not conflated but assessed as distinct therapeutic outcomes (20, 72, 73). The current evidence for lipid-targeted OA interventions, together with the main translational barriers limiting clinical application, is summarized in **Table 2**.

**Table 2:** Lipid-targeted strategies and translational frameworks in osteoarthritis: current evidence and major limitations.

Strategy/translational framework	Representative intervention or target	Evidence base	Main reported benefit or expected value	Major limitation/unresolved issue	Key references
Statins/cholesterol-lowering therapy	HMG-CoA reductase inhibition; statin use	Observational clinical and preclinical evidence	Reduced progression signal in some clinical cohorts; decreased synovial inflammation in preclinical models	Structural benefit remains inconsistent; systemic LDL lowering may not restore tissue-specific lipid handling; therapeutic effects may depend on OA stage, metabolic endotype, and outcome definition	(71, 72)
Enhancing cholesterol efflux	Intracellular cholesterol efflux pathways in chondrocytes	Preclinical mechanistic evidence	Attenuation of OA progression in cartilage-focused preclinical systems	Human translatability and tissue-specific targeting remain unresolved	(47)
Targeting cholesterol-regulatory nodes	CH25H-CYP7B1-RORα; LRP3/Syndecan-4; circARPC1B; SIRT1/FoxO1	Preclinical mechanistic evidence	Reduced catabolic signaling or chondrocyte injury in experimental models	No validated clinical trial framework; pathway specificity and safety remain open questions	(14, 15, 48, 49)
Nutritional fatty-acid intervention	n-3 PUFA supplementation	Clinical and meta-analytic evidence	Improvement in pain and joint function	Evidence for robust structure modification remains weak; response is likely phenotype-dependent	(54–56)
Systemic metabolic intervention in obesity-associated OA	Semaglutide	Clinical trial evidence	Reduced body weight and improved OA-related pain in persons with obesity and knee OA	No evidence of direct cartilage reversal; impact on lipid-defined endotypes remains unknown	(73)
Core non-pharmacologic care	Exercise, education, weight management	Guideline-level clinical recommendation	Remains the standard therapeutic backbone	Not lipid-specific and does not directly test mechanism-based metabolic targeting	(74)
Targeting lipotoxicity, mitochondrial dysfunction, and oxidative stress	Saturated fatty-acid/NOX4/ROS/mtROS/mitophagy-associated pathways	Preclinical mechanistic evidence	Reduced inflammatory signaling, mitochondrial oxidative stress, apoptosis, and ferroptosis susceptibility in chondrocytes	Evidence remains largely cell- or model-based; responder identification and tissue-specific targeting remain unclear	(16, 46, 52, 53, 60, 63, 70)
Targeting neutral-lipid/lipid-droplet metabolism	circRREB1/FASN axis; lipophagy-related pathways	Preclinical mechanistic evidence	Potential attenuation of chondrocyte senescence and restoration of lipid handling	No established clinical intervention; translational biomarkers are lacking	(57–59)
Targeting phospholipid remodeling/ferroptosis	Ferroptosis-associated pathways; phospholipid peroxidation-related processes	Preclinical mechanistic evidence	Attenuation of ferroptotic chondrocyte injury and matrix-destructive responses in experimental models	Human relevance and druggability remain uncertain	(17, 18, 60–64)
Lipidomics-guided biomarker stratification	Circulating lipid profiles; synovial-fluid lipid signatures	Translational biomarker research	May improve phenotyping and define metabolically responsive OA subsets	Blood lipid signatures only partly reflect the joint microenvironment	(17, 19, 20, 76)
Spatial and multi-omics approaches for future trials	Spatial analysis; single-cell atlas; multi-omics integration	Emerging single-cell, spatial, and multi-omics evidence	Tissue-resolved target identification and endotype refinement	Still developmental; requires standardization and prospective validation	(41, 75–78)

This table distinguishes symptomatic benefit, anti-inflammatory effect, and structural disease modification, because these outcomes should not be interpreted as equivalent in osteoarthritis translational research.

HMG-CoA, 3-hydroxy-3-methylglutaryl-coenzyme A; OA, osteoarthritis; PUFAs, polyunsaturated fatty acids; ROS, reactive oxygen species.

### 4.2 Unresolved mechanistic questions in metabolic OA

Several mechanistic questions remain unresolved in metabolic OA (5–7, 25). First, the relationship between circulating lipid abnormalities and tissue-specific pathology is still uncertain (13, 20–22). Clinical and lipidomic studies support an association between metabolic dysfunction and OA severity, yet the variance explained by systemic lipid profiles remains limited, suggesting that blood-based signals do not capture the full biochemical state of the joint (20, 22). Second, the temporal role of lipid dysregulation is not fully defined. Current data do not clearly distinguish whether altered lipid handling acts as an initiating event, a disease amplifier, or a secondary consequence of cartilage and synovial injury. This issue is particularly important when interpreting obesity-related OA, because experimental work indicates that high-fat feeding may prime the joint and the infrapatellar fat pad for more severe pathology after injury, rather than functioning as a sufficient trigger on its own (42). Third, the relevant cellular targets are still being mapped (36–38, 41). Single-cell studies of the infrapatellar fat pad and synovium have shown integrated stromal and inflammatory remodeling, but comparable resolution is still lacking for many lipid-associated pathways in cartilage and subchondral bone (41). Finally, the gap between mechanism and intervention remains substantial. In dyslipidemic mice, cholesterol lowering reduced synovial inflammation without preventing end-stage structural pathology, indicating that modulation of lipid-related inflammation does not automatically translate into disease modification (72). Future studies should therefore test lipid-related mechanisms in defined OA tissues, disease stages, and metabolic phenotypes before extending them to broad causal or therapeutic claims.

### 4.3 Future directions: lipidomics, spatial mapping, and endotype-specific trials

Future progress will depend less on identifying additional lipid associations than on integrating lipid biology into anatomically and clinically meaningful OA frameworks (7, 8, 75, 76, 78). An immediate priority is to combine lipidomics with cell-resolved and spatially resolved approaches (41, 77). Single-cell studies have already shown that the infrapatellar fat pad and synovium form an interactive tissue niche with disease-relevant signaling programs, while spatial analysis has underscored the value of preserving tissue context when interpreting OA microenvironments (41, 77). A second priority is multi-omics integration (8, 76, 78). Recent reviews of OA omics research indicate that transcriptomic, proteomic, metabolomic, and genomic data should not be interpreted in isolation when the goal is to define disease mechanisms rather than descriptive signatures (78). The same principle applies to biomarker development. Future biomarker panels will need to link circulating measures, synovial-fluid signals, and tissue-level biology, with an explicit view toward patient stratification rather than generic prediction (76). This shift is consistent with the growing emphasis on phenotypes, molecular endotypes, and theratypes in OA therapeutic development (75). Clinically, the most informative trials are likely to be those that enroll mechanistically selected subgroups. The semaglutide study in obesity-associated knee OA illustrates that systemic metabolic intervention can improve symptoms in a defined phenotype, but future studies should test whether lipid-centered endotypes can also predict structural or tissue-specific benefit (73).

## 5 Conclusion

Lipid metabolic dysregulation should be regarded as an integral component of OA pathobiology rather than merely as a secondary metabolic by-product of established joint damage (5, 6, 8–10). Current experimental evidence supports six interrelated processes in OA cartilage and related joint tissues: obesity-associated adipose-joint crosstalk, disturbed cholesterol handling, fatty-acid-driven lipotoxicity and mitochondrial oxidative injury, abnormal neutral-lipid storage with impaired disposal, phospholipid remodeling linked to ferroptotic vulnerability, and context-dependent HIF-1α-centered metabolic adaptation (14–18, 52, 53, 57–59, 63, 70). These processes do not occur in isolation, because cartilage biology is influenced by crosstalk with synovium, subchondral bone, and the infrapatellar fat pad within a whole-joint metabolic microenvironment (1–3, 28, 29, 38). As summarized in **Figure 1**, these processes converge on recurrent pathological outputs, including inflammatory amplification, chondrocyte senescence, apoptosis, ferroptosis susceptibility, and extracellular matrix degradation.

At the clinical level, the available data support the relevance of metabolic stratification in OA, but they do not yet justify the claim that any single lipid-targeted strategy has been proven to modify disease progression broadly across patient populations (71–73). Future studies should test defined lipid pathways against OA phenotypes and therapeutic responses using primary mechanistic evidence and biomarker-guided study designs (7, 10, 20, 22, 75).
